# Supplementary material for: Identification of photoperiod-induced specific miRNAs in the adrenal glands of Sunite sheep (Ovis aries)
Source: Front Vet Sci. 2022 Jul 22;9:888207. doi: 10.3389/fvets.2022.888207 (PMC9354845; doi:10.3389/fvets.2022.888207)
Supplement: Supplementary file 1 [file Data_Sheet_1.ZIP › Supplementary file/miRDeep2 file.zip!/miRDeep2 file/Know_Structure/SP42A1/oar-mir-106b.pdf]

miRBase precursor : oar-mir-106b
Total read count : 3351
oar-miR-106b read count : 1486
remaining reads : 1865

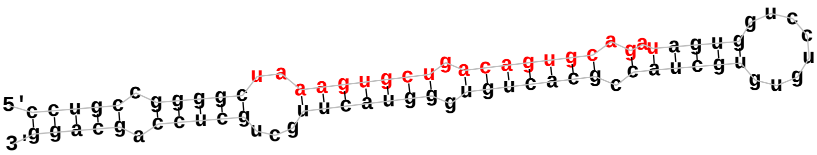

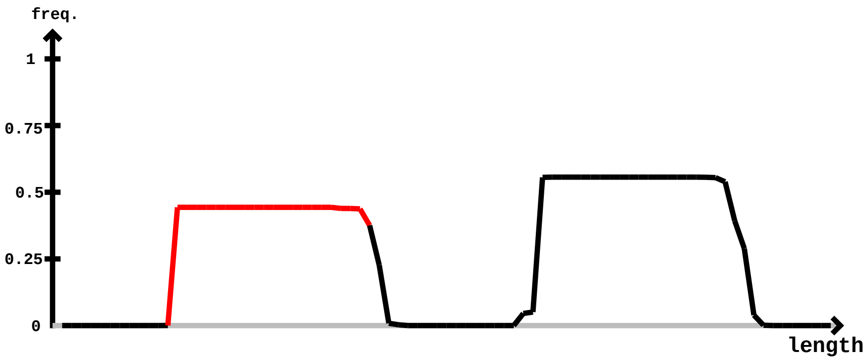


```
                    oar-miR-106b
5'- ccugccggggcuaaagugcugacagugcagauagugguccugugugcuaccgcacuguggguacuugcugcuccagcagg   -3'   exp
    (((((.(((((..(((((((.(((((((.(.(((((........)))))).))))))).)))))))...))))).)))))    reads   mm   sample
    ..........cuaaagugcugacagugcagau................................................    1       0    seq
    ...........uaaagugcugacagugc....................................................    12      0    seq
    ...........uaaagugcugacagugA....................................................    1       1    seq
    ...........uaaaAugcugacagugc....................................................    1       1    seq
    ...........uaaagugcugacagugca...................................................    1       0    seq
    ...........uaaagugcugacagugcag..................................................    5       0    seq
    ...........uaaagugcugacagugcaga.................................................    206     0    seq
    ...........uaaagugcugacagugcaCa.................................................    1       1    seq
    ...........uaaagugcugacagugcagau................................................    498     0    seq
    ...........uaaagugcugacagugcagaua...............................................    731     0    seq
    ...........uaaagugcugacagugcagauag..............................................    19      0    seq
    ...........uaaagugcugacagugcagauagu.............................................    10      0    seq
    ...............................................uaccgcacuguggguacuu..............    1       0    seq
    ...............................................uaccgcacuguggguacuug.............    1       0    seq
    ...............................................uaccgcacuguggguacuugc............    13      0    seq
    ...............................................uaccgcacuguggguacuugcu...........    133     0    seq
    ...............................................uaccgcacuguggguacuugcug..........    2       0    seq
    ...............................................uaccgcacuguggguacuugcugc.........    2       0    seq
    ................................................accgcacuguggguacuugcu...........    12      0    seq
    ................................................accgcacuguggguacuugcug..........    5       0    seq
    ................................................accgcacuguggguacuugcugc.........    1       0    seq
    .................................................ccgcacuguggguacuu..............    1       0    seq
    .................................................ccgcacuguggguacuug.............    3       0    seq
    .................................................ccgcacuguggguacuugc............    39      0    seq
    .................................................ccgcacuguggguacuugcu...........    344     0    seq
    .................................................ccgcacuguggguacuugcug..........    342     0    seq
    .................................................ccgcacuguggguacuugcugc.........    832     0    seq
    .................................................ccgcacuguggguacuugcugcu........    127     0    seq
    .................................................ccgcacuguggguacuugcugcuc.......    4       0    seq
    ..................................................cgcacuguggguacuugcu...........    1       0    seq
    ..................................................cgcacuguggguacuugcugc.........    2       0    seq
```
